# Real-world national trends and socio-economic factors preference of sodium-glucose cotransporter-2 inhibitors and glucagon-like peptide-1 receptor agonists in China

**DOI:** 10.3389/fendo.2022.987081

**Published:** 2022-10-07

**Authors:** Cao Li, Shanshan Guo, Jiping Huo, Yiming Gao, Yilong Yan, Zhigang Zhao

**Affiliations:** ^1^ Department of Pharmacy, Beijing Tiantan Hospital, Capital Medical University, Beijing, China; ^2^ Department of Clinical Pharmacology, School of Pharmaceutical Sciences, Capital Medical University, Beijing, China

**Keywords:** SGLT2i, GLP-1RA, type 2 diabetics, cardiovascular diseases, chronic kidney disease

## Abstract

**Backgrounds:**

Robust evidence have demonstrated the beneficial effect of Sodium-glucose cotransporter-2 inhibitors (SGLT2i) and glucagon-like peptide-1 receptor agonists (GLP-1RA) in T2D patients with cardiovascular diseases and chronic kidney disease. Multiple studies analyzed patterns and predictors of SGLT2i and GLP-1RA in the US, Europe and worldwide. However, there is no study about the utilization of these two classes of drugs in real-world in China.

**Method:**

A total of 181743 prescriptions of SGLT2i and 59720 GLP-1RA were retrospectively pooled from Hospital Prescription Analysis Cooperation Project from 2018 to 2021. The social-economic characteristics of patients and prescribers, including age, gender, residency, hospital level, insurance type, department visited, and payment amount, were collected and analyzed to study trends and risk factors associated with preference among two antidiabetics.

**Results:**

Annual number of prescriptions of SGLT2i significantly increased to approximately 140 folds, while GLP-1RA increased to about 6.5 folds. After adjustment for socio-economic information, several patients or physician characteristics were positively associated with the preference of GLP-1RA, including female gender (OR 1.581, 95% CI 1.528-1.635), residents in second-tier cities (OR 1.194, 95% CI 1.148-1.142), visiting primary or secondary hospital level (OR 2.387, 95% CI 2.268-2.512); while other factors were associated with the preference of SGLT2i, including older adults (OR 0.713, 95% CI 0.688-0.739), uncovered by insurance (OR 0.310, 95% CI 0.293-0.329), visiting other departments compared with endocrinology. In addition, the share of SGLT2i and GLP-1RA was low but in an increasing tendency.

**Conclusions:**

SGLT2i and GLP-1RA prescription significantly increased from 2018 to 2021. The socio-economic risk factors in choosing SGLT2i or GLP-1RA highlight an effort required to reduce disparities and improve health outcomes.

## Introduction

With the development of economy and the growth of urbanization, the global prevalence of diabetes was approximately 10.5% in 2021 and is estimated to rise to 12.2% in 2045, resulting in serious health consequences and socio-economic problems (1). Patients with type 2 diabetes (T2D) are at elevated risk of cardiovascular disease (CVD) and chronic kidney diseases (CKD), which are the leading cause of morbidity and mortality (2). Recently several randomized controlled trials have demonstrated that two classes of glucose-lowering medications, glucagon-like peptide-1 receptor agonists (GLP-1RA) and sodium-glucose cotransporter-2 inhibitors (SGLT2i), have shown a significant protective effect on CVD or CKD in T2D patients (3–5). In addition, guidelines of the European Association for the Study of Diabetes (EASD) and the American Diabetes Association (ADA) have updated the major role of both SGLT2i and GLP-1RA in the management of T2D patients with reno- or cardio- complications (6).

As the approval of new classes of medications, pharmacotherapy of T2D has changed remarkably over the past 2 decades. For instance, monotherapy of GLP-1RA and SGLT2i increased to approximately 2-3 folds from 2015 to 2019 in the United States (7). A similar substantially increasing of these two medications was found in Europe, and the change even occurred before the published guidelines (8, 9). Although the new agent SGLT2i and GLP-1RA were already widely used globally, large country differences still exist due to the characteristics of patients and the capacity of financial systems (10).

In China, the prevalence of diabetic adults was estimated as 12.4% and higher than the global average (8.3%) (11). Importantly, the prevalence of adequate management was not significantly improved. The first GLP-1RA and SGLT2i were incorporated into the national medical insurance reimbursement list in late 2017 and 2020, respectively. To the best of our knowledge, large-scale study of these two classes of antidiabetics was not reported yet in China. Thus, the present study aims (1) to study prescription trends of GLP-1RA and SGLT2i; (2) to characterize socio-economic factors associated with the selection of these two classes of drugs from 2018 to 2021 in real-world.

## Methods

### Data sources

This study was designed as a retrospective cross-sectional study based on prescription data. Prescriptions were extracted from the Cooperation Project Database of Hospital Prescriptions (CPDHP), which has been extensively used for pharmacoepidemiology studies in China (12, 13). The CPDHP is a multi-institutional database consisting of prescription information obtained from 102 hospitals in 9 cities or provinces, including Shanghai, Hangzhou, Beijing, Tianjin, Shenyang, Zhengzhou, Harbin, Chengdu, and Guangzhou. A random sample of 10-day prescriptions in the above hospitals is selected quarterly each year, containing two non-consecutive Mondays to Fridays (except national holidays). Information in prescriptions were collected and unified in same format, including the sample city/province, patient number, gender, age, department, visit time, insurance type, generic drug name, hospital level, and payment amount. Furthermore, the personal information of patients and clinicians were masked in this project. The present study was approved by the ethics committee at Beijing Tiantan Hospital, Capital Medical University.

### Study sample

In the present study, outpatient prescriptions that met the following criteria were included: (1) prescribed for patients aged over 18 years; (2) containing at least one SGLT2i or GLP-1RA; and (3) prescribed between 2018 and 2021. However, the prescriptions with missing age or sex were excluded.

### Drug classes

Antidiabetics in this study were coded according to the World Health Organization Anatomical Therapeutic Chemical classification system, which were divided into two categories: (1) A10BJ, GLP-1RA: Exenatide, Liraglutide, Lixisenatide, Dulaglutide, Semaglutide, Beinaglutide, and Loxenatide; (2) A10BK, SGLT2i: Dapagliflozin, Canagliflozin, Empagliflozin, and Ertugliflozin; this study only focuses on these two classes of antidiabetic agents.

### Statistical analysis

Relative numbers of medications per year were calculated with the number of annual total prescriptions. Data were presented as means ± SD for continuous variables or as frequency (%) for categorical variables. Baseline characteristics between SGLT2i and GLP-1RA groups were analyzed using χ2 tests. A logistic regression model was performed to analyze patient and prescriber socio-economic characteristics to analyze preference of SGLT2i or GLP-1RA. All data were conducted and analyzed using Microsoft Excel and IBM SPSS Statistics (version 26; IBM Corporation, USA).

## Results

### Overall trends in utilization of SGLT2i and GLP-1RA

**Figure 1:**
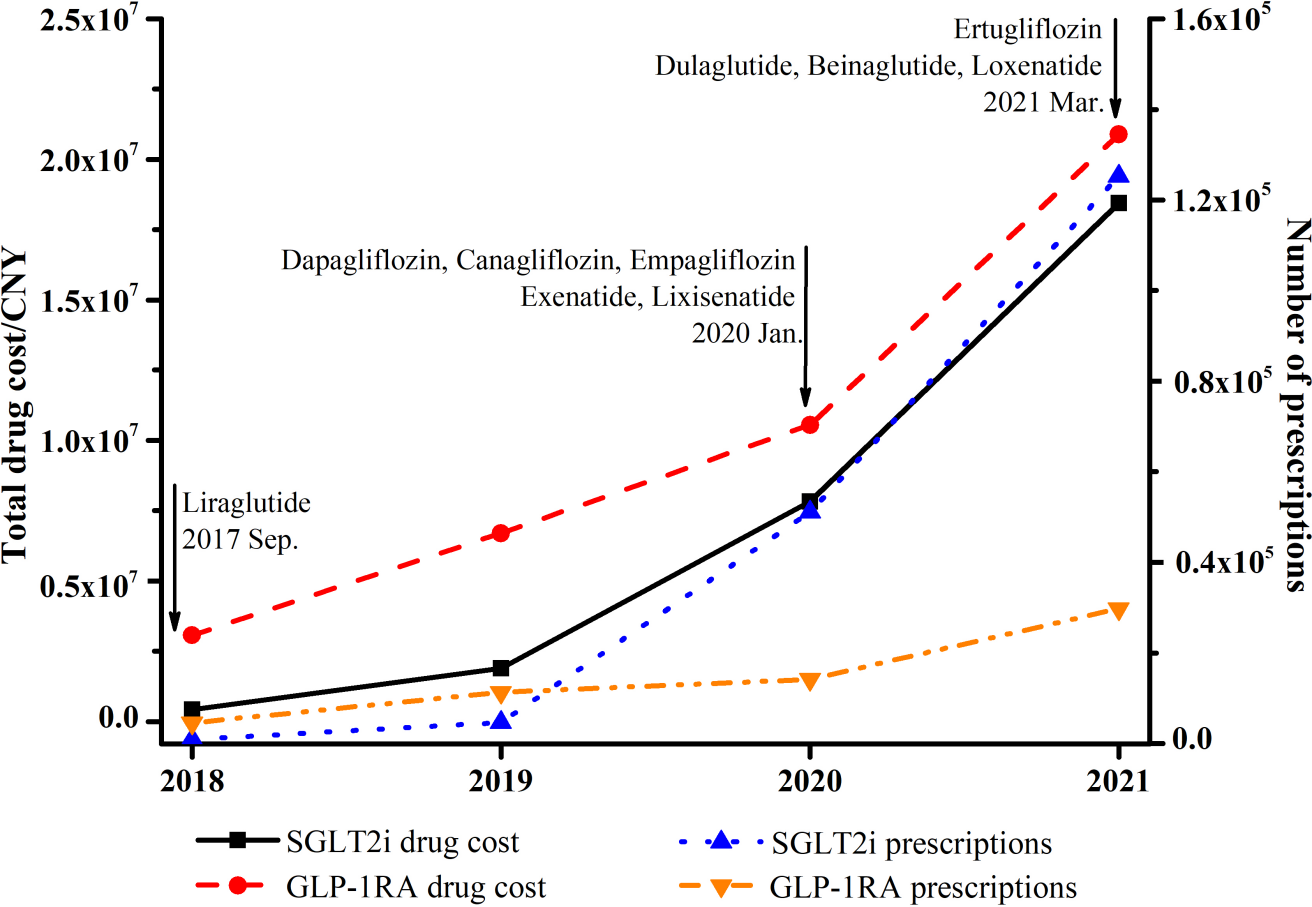
Total trends of expenditure and prescriptions of SGLT2i and GLP-1RA from 2018 to 2021.

**Figure 2:**
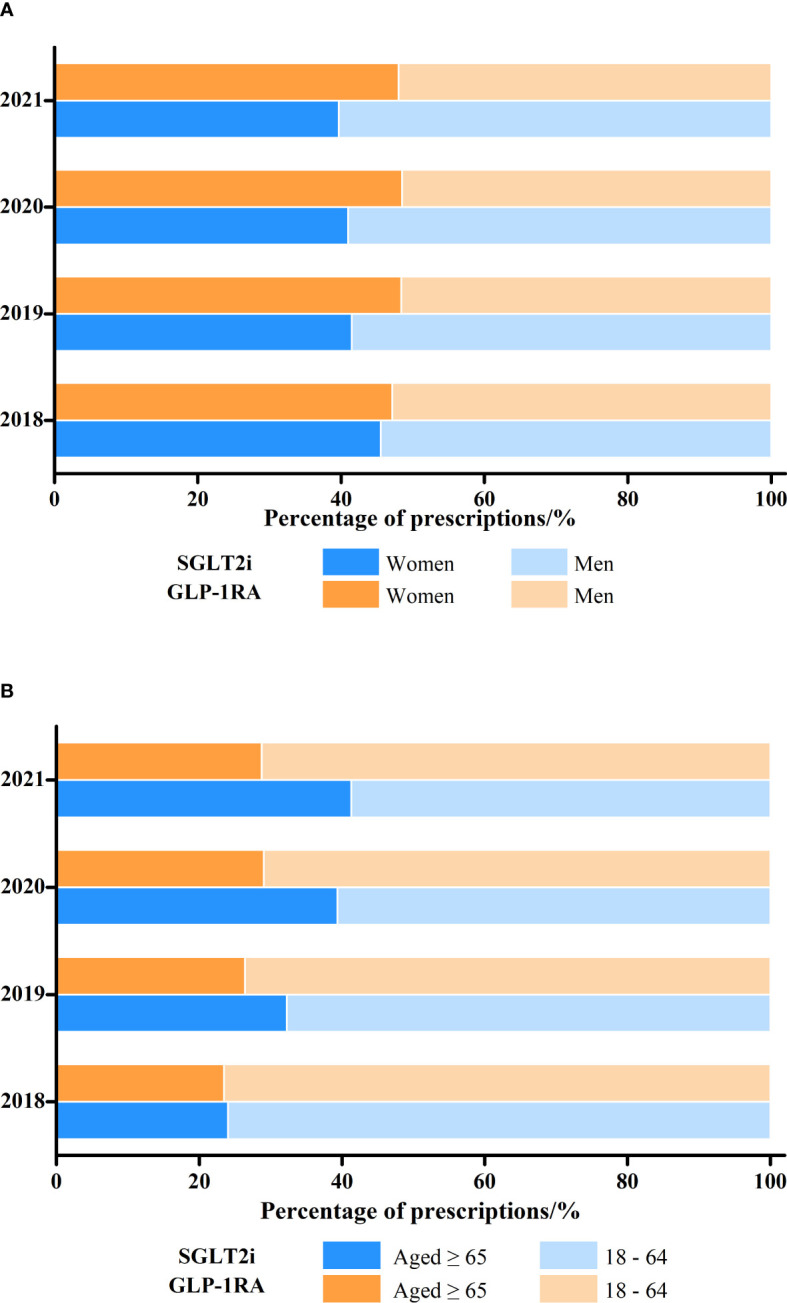
Changes in the proportion of SGLT2i and GLP-1RA prescriptions dispensed by **(A)** patient gender and **(B)** age, 2018-2021.

A total of 181743 prescriptions of SGLT2i and 59720 GLP-1RA were pooled from the outpatient of CPDHP database (**Table 1**). The annual number of prescriptions of SGLT2i dramatically increased to approximately 140 folds, while GLP-1RA increased to about 6.5 folds, from 2018 to 2021 (**Figure 1**; **Table 1**). With respect to the total annual medication expenditure of these two classes of antidiabetics, it showed a similar tendency with growth to 44 and 6.8 folds, respectively. In addition, the trends of gender and age in drug utilization were also analyzed. The proportion of females using SGLT2i decreased considerably from 45.5% in 2018 to 39.6% in 2021, while the use of GLP-1RA was steady (**Figure 2A**; **Supplementary Tables S1, S2**). The proportion of SGLT2i and GLP-1RA used in subjects aged 18-64 both decreased in the study period, from 76.0% to 58.7% and from 76.5% to 71.3% (**Figure 2B**; **Supplementary Tables S1, S2**).

**Table:** Table 1 Baseline characteristics of the study sample.

Characteristic	SGLT2i	GLP-1RA	P-value
**Total**	181743 (100)	59720 (100)	
**Gender**			0.001
Male	108893 (59.9)	30988 (51.9)	
Female	72850 (40.1)	28732 (48.1)	
**Age** (mean, SD)			0.001
18-64	52.5 (9.2)	48.3 (11.1)	
≥65	72.8 (6.6)	72.1 (6.4)	
**Year**			<0.001
2018	899 (0.5)	4539 (7.6)	
2019	4597 (2.5)	11271 (18.9)	
2020	51035 (28.1)	14157 (23.7)	
2021	125212 (68.9)	29753 (49.8)	
**City Type**			<0.001
First -tier	126467 (69.6)	38057 (63.7)	
Other	55276 (30.4)	21663 (36.3)	
**Hospital Level**			<0.001
Tertiary hospitals	165680 (91.2)	53503 (89.6)	
Primary or secondary	16063 (8.2)	6217 (10.4)	
**Insurance Type**			<0.001
Full or partial	133512 (73.5)	46249 (77.5)	
Self-pay	31830 (17.5)	6516 (10.9)	
Unknown	16401 (9.0)	6935 (11.6)	
**Department Visited**			<0.001
Endocrinology	119071 (65.5)	50867 (85.2)	
Cardiology	19452 (10.7)	1437 (2.4)	
Nephrology	5907 (3.3)	428 (0.7)	
GP/IM	20650 (11.4)	2890 (4.8)	
Others	16663 (9.2)	4098 (6.9)	
**Payment (mean, SD)**	157.2 (106.7)	690.0 (488.8)	<0.001

### Characteristics of study population using SGLT2i and GLP-1RA

Characteristics of subjects who used SGLT2i or GLP-1RA from 2018 to 2021 are presented in **Table 1**. The female gender was more likely to be prescribed GLP-1RA (48.1 vs 40.1%, *p* = 0.001) than SGLT2i. Subjects treated with SGLT2i were significantly older than those treated with GLP-1RA in both groups aged ≥65 and 18-64 (**Table 1**). The majority of patients are residents in robust economic first-tier cities of Beijing, Shanghai, and Guangzhou (**Tables 1**; **Supplementary Tables S3, S4**). Most SGLT2i (91.2%) and GLP-1RA (89.6%) were provided by tertiary hospitals other than primary or secondary ones. In terms of department/clinician visited, most patients received prescription from endocrinologists, cardiologists, nephrologists, general partitioners/internal medical (GP/IM) physicians (SGLT2i, 65.5%, 10.7%, 3.3%, 11.4%; GLP-1RA, 85.2%, 2.4%, 0.7%, 4.8%), respectively. In addition, approximately three-quarters population were covered by full or partial insurance, although some insurance information was missing.

### Associated factors of selection on SGLT2i or GLP-1RA

To better understand associated socio-economic factors in choosing SGLT2i or GLP-1RA, the logistic regression model was used to determine the probable predictors (**Table 2**). Using adults aged from 18-64 as a reference, the odds ratio for SGLT2i (OR 0.713, 95% CI 0.688-0.739) increased along with age compared with GLP-1RA. The probability of using SGLT2i was lower in female (OR 1.581, 95% CI 1.528-1.635) gender than in male. Among the study periods, we found less use of GLP-1RA in 2019 (OR 0.618, 95% CI 0.561-0.681), 2020 (OR 0.451, 95% CI 0.410-0.495), and 2021 (OR 0.474, 95% CI 0.432-0.519) compared with 2018. In addition, self-pay patients had a significantly lower prevalence of using GLP-1RA (OR 0.310, 95% CI 0.293-0.329); in contrast, patients visiting primary or secondary hospitals (OR 2.387, 95% CI 2.268-2.512) had a significantly higher chance of receiving GLP-1RA.

**Table:** Table 2 The social-economic factors associated with selection of SGLT2i or GLP-1RA.

Characteristic	OR	95% CI	*P*
**Gender**			
Female	1.581	1.528-1.635	< 0.001
**Age**			
≥65	0.713	0.688-0.739	< 0.001
**Year**			
2018	ref		
2019	0.618	0.561-0.681	< 0.001
2020	0.451	0.410-0.495	< 0.001
2021	0.474	0.432-0.519	< 0.001
**City Type**			
First -tier	ref		
Other	1.194	1.148-1.242	< 0.001
**Hospital Level**			
Tertiary hospitals	ref		
Primary or secondary	2.387	2.268-2.512	< 0.001
**Insurance Type**			
Full or partial	ref		
Self-pay	0.310	0.293-0.329	< 0.001
Unknown	0.505	0.476-0.537	< 0.001
**Department Visited**			
Endocrinology	ref		
Cardiology	0.109	0.099-0.120	< 0.001
Nephrology	0.306	0.261-0.358	< 0.001
GP/IM	0.146	0.133-0.155	< 0.001
Others	0.612	0.575-0.651	< 0.001
**Payment Amount**	1.015	1.015-1.016	< 0.001

"ref" is abbreviated for reference.

**Figure 3:**
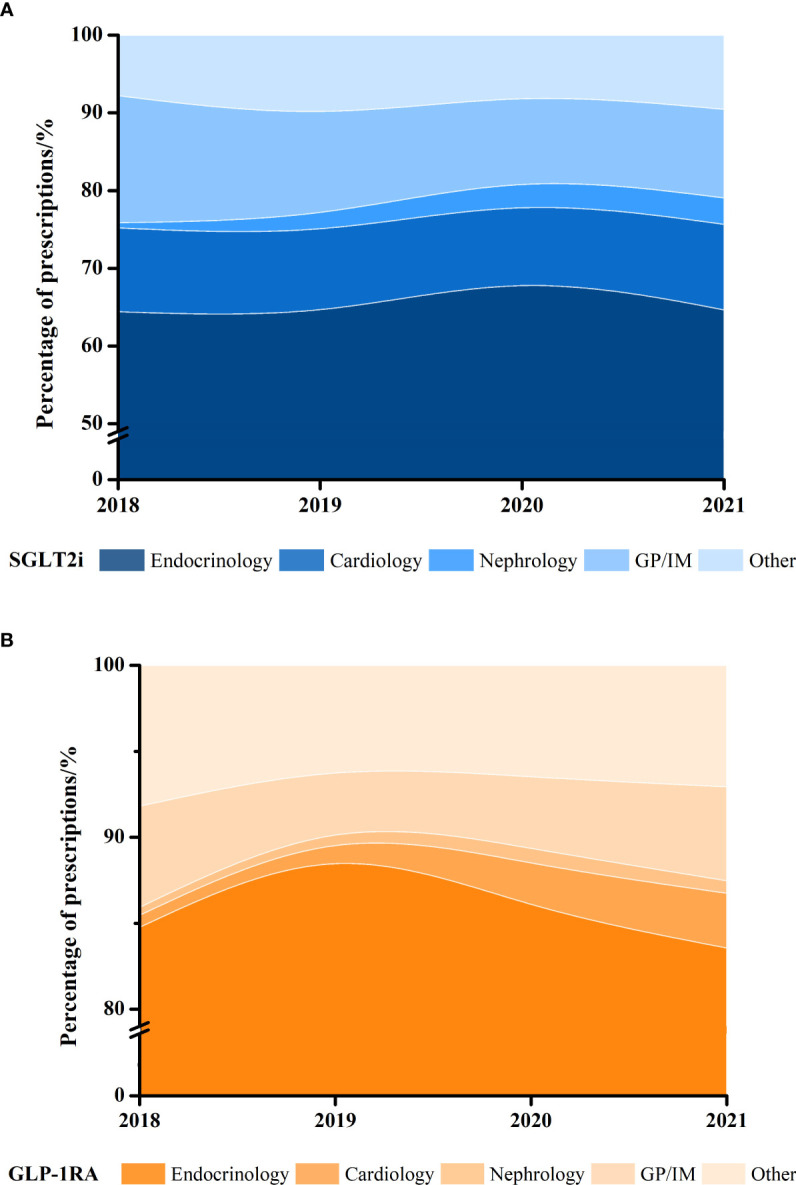
Proportion of yearly **(A)** SGLT2i and **(B)** GLP-1RA dispensed prescriptions in different clinical departments, 2018–2021.

Compared with endocrinologists, all other specialties including in cardiology (OR 0.109, 95% CI 0.099-0.120), nephrology (OR 0.306, 95% CI 0.261-0.358), GP/IM (OR 0.146, 95% CI 0.133-0.155) had a lower preference prescribing GLP-1RA to patients (**Table 2**). Worthy mentioning, although the proportion of two classes of drugs was low in the department of cardiology and nephrology, it showed an overall increasing tendency with SGLT2i (nephrology, 0.7% to 3.4%) and GLP-1RA from 2018 to 2021 (nephrology, 0.5% to 0.7%; cardiology, 0.7% to 3.2%), except stable tendency of SGLT2i in cardiology (10.8% to 11.0%, **Figure 3**; **Supplementary Tables S1, S2**).

## Discussion

To the best of our knowledge, this is the first nationwide representative retrospective analysis of new cardio-reno beneficial antidiabetics in the real-world in mainland China. Our study comprehensively showed that (1) a substantial increase in the utilization of SGLT2i (140 folds) and GLP-1RA (6.5 folds) from 2018 to 2021 (**Figure 1**; **Table 1**); (2) various pharmacoeconomic and prescriber characteristics associated with the preference of SGLT2i or GLP-1RA: male, and self-pay patients are more likely to be treated with SGLT2i; while healthcare clinicians in primary or secondary hospitals, younger adults, endocrinologists prefer to prescribe GLP-1RA over SGLT2i (**Table 2**); (3) absolute number of prescriptions and proportion of new antidiabetics showed an overall increasing trend in the department of cardiology and nephrology (**Figure 3**; **Supplementary Tables S1, S2**).

A significant growth in the utilization and drug expenditure of SGLT2i and GLP-1RA was found in the 4-year study period. First of all, the decision of the National Medical Products Administration (NMPA) and the National Healthcare Security Administration (NHSA) had a substantial influence on the medication trends (**Figure 1**). After new antidiabetics GLP-1RA and SGLT2i were approved by NMPA, the first GLP-1RA (2017 Sep.) and SGLT2i (2020 Jan.) were reimbursed by national insurance lists in China. The overall trend is similar compared to the US (14), Europe (9), and other countries or continents worldwide (10) in previous studies. However, there were still some differences between China and US. For instance, increasing utilization of GLP-1RA was steeper and dramatic in China from 2018 (2nd year covered by national insurance) to 2021, compared with 2008 (2nd year included in Medicare) to 2011 (15, 16), this might be due to GLP-1RA was confirmed beneficial to CVDs patients was progressively proved in 2015 (17, 18). Second, all authoritative western and China guidelines (19–22) have strongly recommended the application of SGLT2i and GLP-1RA in diabetic patients with CVD complications. As heart failure indication of SGLT2i approved in 2022, presumable indication expansion of GLP-1RA, a predicable major increasing share of new antidiabetics and structure change of antihyperglycemic medication may take place in following years.

Our study revealed gender and age differences in the selection of new antidiabetics. The present study indicates that the female gender was more likely to be treated with GLP-1RA, and a prior study suggested that female patients had a higher chance of receiving GLP-1RA (23). The potential mechanism could be the utilization of GLP-1RA in gestational diabetes mellitus, a disease only harms women. Studies have indicated a potential beneficial effect of GLP-1RA on the female with different stages of gestational diabetes mellitus (24–26). In addition, polycystic ovary syndrome (PCOS), a common disease affecting up to 20% of women of reproductive age, was shown to be alternatively alleviated by GLP-1RA with clinical evidence (15, 27–29). Physiologically, GLP-1RA modulates mammalian hypothalamus, pituitary, gonads, ovaries, and has an anti-inflammatory and anti-fibrotic function. In the aspect of age, older adults had a higher chance of being prescribed SGLT2i. Clinical guidelines for elderly T2D in China recommend an escalation therapy for pharmacotherapy, and according to guideline for monotherapy of SGLT2i has no risk of hypoglycemia while GLP-1RA does not (30). Generally, older adults are more vulnerable to hypoglycemia, a condition sometimes even life-threatening. In addition, SGLT2i has one more recommendation of treating heart failure compared with GLP-1RA in older adults, and SGLT2i was showed more beneficial in renal outcomes (30, 31).

Economic factors could have a substantial influence on the selection of medications. Our results suggest that subjects without insurance coverage, and visiting tertiary hospitals were more frequently prescribed SGLT2i than GLP-1RA. The average payment of GLP-1RA (690.0 ± 488.8 CNY) was significantly higher than SGLT2i (157.2 ± 106.7 CNY). In the US, the relative high-cost affects that the new antidiabetics are most frequently prescribed to patients with private insurance and least to people with self-pay (32). The hospital level was classified as another economic factor, because the China health insurance system is different from developed western countries. Actually, the floating population, whom originally living in less developed cities or rural areas, leaving their hometown, working or seeking medical services in tertiary hospitals located in large first tier cities, normally has a lower income and more sensitive to drug price; whereas urban-resident patients visiting primary or secondary may have a better income status and financial affordability. Thus, the patient in different-tier cities might reflects the financial conditions. To be mentioned, although T2D prevalence was higher in urban cities, patients living in rural areas had excess mortality (33, 34). Higher costs might be barriers to dispensing new classes of antidiabetics for vulnerable patients, which might further lead to medical inequality (35, 36).

Clinicians in different departments play an important role in the selection of new hyperglycemia medications. Similar with prior studies (14, 37), our study suggests that cardiologists or nephrologists prefer prescribing SGLT2i over GLP-1RA compared to endocrinologists (**Table 2**). According to EASD/ADA guideline (38), SGLT2i is preferably recommended for reducing HF and/or CKD progression in cardiovascular outcomes trials (CVOTs), which could be the potential reason for more favorable in the department of cardiology and nephrology. As GLP-1RA was recently approved for treating obesity and evidences showed an effect of losing weight, overweight or obesity patients may visit department of endocrinology, and then treated by GLP-1RA. The substantial growth of SGLT2i and GLP-1RA was observed similarly in our study and US (37, 39); however, most prescriptions were dispensed in the department of endocrinology in China, but primary care/internal medicine physicians in the US. In addition, although robust evidence and guidelines have recommended new antidiabetics, patients meeting appropriate criteria still were not treated with them. Concerning one-third of T2D patients concomitant with CVD (40, 41) and almost 40% concomitant with CKD (16), the low prevalence of SGLT2i and GLP-1RA in endocrinology and nephrology makes them important candidates for significant clinical benefits.

In conclusion, the present nationwide real-world study highlights that SGLT2i and GLP-1RA prescription significantly increased from 2018 to 2021, which may contribute to substantial welfare for T2D patients with CVD or CKD. In addition, our results showed some patient socio-economic and prescriber characteristics are associated with the preference of SGLIT2i over GLP-1RA, including male gender, relative older age, patients vising tertiary hospitals, uncovered by insurance and department other than endocrinology, which means there is still a lot of work to reduce disparities, guarantee patient safety and improve health outcomes.

Our study had some limitations. First, the present study only analyzed SGLT2i and GLP-1RA, and other information of antidiabetic medications was missing; it is unable to study the overall structure change of diabetic pharmacotherapy. Second, although the present study included a large number of cross-sectional samples, it should be cautious to apply the current conclusion to the general Chinese population, especially in less developed cities and rural areas. Third, since the co-prescribing information of medications and laboratory tests (glucose, HbA1c, *etc.*) was not accessed, it is unable to evaluate and track therapeutic and adverse effects.

## Data availability statement

The original contributions presented in the study are included in the article/**Supplementary Material**. Further inquiries can be directed to the corresponding author.

## Ethics statement

The studies involving human participants were reviewed and approved by Beijing Tiantan Hospital, Capital Medical University. Written informed consent for participation was not required for this study in accordance with the national legislation and the institutional requirements.

## Author contributions

CL, SG and ZZ conceived and designed the study. CL and SG wrote the manuscript. CL, SG, JH, YG, YY, and ZZ performed statistics and generated the figures and tables. All authors contributed to the revision of paper. All authors listed have made a direct and substantial contribution to the manuscript, and approved it for publication.

## Funding

This work was supported by MiaoPu Project of Beijing Tiantan Hospital (2020MP07); Yangfan Project of Beijing Hospitals Authority (ZYLX201827); the National Key Research and Development Program (2016YFE0205400).

## Conflict of interest

The authors declare that the research was conducted in the absence of any commercial or financial relationships that could be construed as a potential conflict of interest.

## Publisher’s note

All claims expressed in this article are solely those of the authors and do not necessarily represent those of their affiliated organizations, or those of the publisher, the editors and the reviewers. Any product that may be evaluated in this article, or claim that may be made by its manufacturer, is not guaranteed or endorsed by the publisher.
