# Supplementary material for: Real-world national trends and socio-economic factors preference of sodium-glucose cotransporter-2 inhibitors and glucagon-like peptide-1 receptor agonists in China
Source: Front Endocrinol (Lausanne). 2022 Oct 7;13:987081. doi: 10.3389/fendo.2022.987081 (PMC9585197; doi:10.3389/fendo.2022.987081)
Supplement: Supplementary file 1 [file Table_1.docx]

**SUPPLEMENTARY MATERIAL**

**Supplementary Table S1 |** SGLT2i prescriptions dispensed by patient characteristics and clinical department, 2018-2021 [N (%)].

| **Characteristics** | **2018** | **2019** | **2020** | **2021** | **Total from 2018-2021** |
| --- | --- | --- | --- | --- | --- |
| **Patient Gender** | | | | | |
| Male | 490 (54.5) | 2691 (58.5) | 30146 (59.1) | 75566 (60.4) | 108893 (59.9) |
| Female | 409 (45.5) | 1906 (41.5) | 20889 (40.9) | 49646 (39.6) | 72850 (40.1) |
| **Patient Age** |  |  |  |  |  |
| 18-64 | 683 (76.0) | 3115 (67.8) | 30953 (60.7) | 73529 (58.7) | 108280 (59.6) |
| ≥65 | 216 (24.0) | 1482 (32.2) | 20082 (39.3) | 51683 (41.3) | 73463 (40.4) |
| **Department Visited** | | | | | |
| Endocrinology | 579 (64.4) | 2973 (64.7) | 34576 (67.7) | 80943 (64.6) | 119071 (65.5) |
| Cardiology | 97 (10.8) | 479 (10.4) | 5116 (10.0) | 13760 (11.0) | 19452 (10.7) |
| Nephrology | 6 (0.7) | 96 (2.1) | 1532 (3.0) | 4273 (3.4) | 5907 (3.3) |
| GP/IM | 147 (16.4) | 597 (13.0) | 5631 (11.0) | 14275 (11.4) | 20650 (11.4) |
| Others | 70 (7.8) | 452 (9.8) | 4180 (8.2) | 11961 (9.6) | 16663 (9.2) |
| **Total prescriptions** | 899 (100.0) | 4597 (100.0) | 51035 (100.0) | 125212 (100.0) | 181743 (100.0) |

**Supplementary Table S2 |** GLP-1RA prescriptions dispensed by patient characteristics and clinical department, 2018-2021 [N (%)].

| **Characteristics** | **2018** | **2019** | **2020** | **2021** | **Total from 2018-2021** |
| --- | --- | --- | --- | --- | --- |
| **Patient Gender** | | | | | |
| Male | 2400 (52.9) | 5820 (51.6) | 7296 (51.5) | 15472 (52.0) | 30988 (51.9) |
| Female | 2139 (47.1) | 5451 (48.4) | 6861 (48.5) | 14281 (48.0) | 28732 (48.1) |
| **Patient Age** | | | | | |
| 18-64 | 3474 (76.5) | 8294 (73.6) | 10045 (71.0) | 21205 (71.3) | 43018 (72.0) |
| ≥65 | 1065 (23.5) | 2977 (26.4) | 4112 (29.1) | 8548 (28.7) | 16702 (28.0) |
| **Department Visited** | | | | | |
| Endocrinology | 3847 (84.8) | 9970 (88.5) | 12189 (86.1) | 24861 (83.6) | 50867 (85.2) |
| Cardiology | 32 (0.7) | 119 (1.1) | 341 (2.4) | 945 (3.2) | 1437 (2.4) |
| Nephrology | 21 (0.5) | 69 (0.6) | 120 (0.8) | 218 (0.7) | 428 (0.7) |
| GP/IM | 267 (5.9) | 407 (3.6) | 590 (4.2) | 1626 (5.5) | 2890 (4.8) |
| Other | 372 (8.2) | 706 (6.3) | 917 (6.5) | 2103 (7.1) | 4098 (6.9) |
| **Total prescriptions** | 4539 (100.0) | 11271 (100.0) | 14157 (100.0) | 29753 (100.0) | 59720 (100.0) |

**Supplementary Table S3 |** Percentage of SGLT2i in different cities/provinces [N (%)].

|  | **2018** | **2019** | **2020** | **2021** | **Total from 2018-2021** |
| --- | --- | --- | --- | --- | --- |
| Shanghai | 186 (20.7) | 684 (14.9) | 14827 (29.1) | 33013 (26.4) | 48710 (26.8) |
| Guangzhou | 283 (31.5) | 1810 (39.4) | 13769 (27.0) | 30597 (24.4) | 46459 (25.6) |
| Beijing | 47 (5.2) | 463 (10.1) | 8236 (16.1) | 22552 (18.0) | 31298 (17.2) |
| Hangzhou | 0 (0.0) | 62 (1.4) | 3549 (7.0) | 13466 (10.8) | 17077 (9.4) |
| Chengdu | 0 (0.0) | 252 (5.5) | 3226 (6.3) | 8019 (6.4) | 11497 (6.3) |
| Zhengzhou | 230 (25.6) | 909 (19.8) | 3446 (6.8) | 5920 (4.7) | 10505 (5.8) |
| Shenyang | 137 (15.2) | 218 (4.7) | 1678 (3.3) | 6585 (5.3) | 8618 (4.7) |
| Tianjin | 1 (0.1) | 47 (1.0) | 1289 (2.5) | 3276 (2.6) | 4613 (2.5) |
| Harbin | 15 (1.7) | 152 (3.3) | 1015 (2.0) | 1784 (1.4) | 2966 (1.6) |
| ALL | 899 (100.0) | 4597 (100.0) | 51035 (100.0) | 125212 (100.0) | 181743 (100.0) |

**Supplementary Table S4 |** Percentage of GLP-1RA in different cities/provinces [N (%)].

|  | **2018** | **2019** | **2020** | **2021** | **Total from 2018-2021** |
| --- | --- | --- | --- | --- | --- |
| Shanghai | 1934 (42.6) | 4658 (41.3) | 5547 (39.2) | 10693 (35.9) | 22832 (38.2) |
| Beijing | 407 (9.0) | 1056 (9.4) | 1584 (11.2) | 5610 (18.9) | 8657 (14.5) |
| Tianjin | 255 (5.6) | 2077 (18.4) | 2038 (14.4) | 3079 (10.4) | 7449 (12.5) |
| Guangzhou | 499 (11.0) | 1066 (9.5) | 1598 (11.3) | 3405 (11.4) | 6568 (11.0) |
| Hangzhou | 470 (10.4) | 929 (8.2) | 1192 (8.4) | 2750 (9.2) | 5341 (8.9) |
| Shenyang | 406 (8.9) | 372 (3.3) | 648 (4.6) | 1401 (4.7) | 2827 (4.7) |
| Chengdu | 157 (3.5) | 377 (3.3) | 612 (4.3) | 1243 (4.2) | 2389 (4.0) |
| Zhengzhou | 364 (8.0) | 498 (4.4) | 564 (4.0) | 841 (2.8) | 2267 (3.8) |
| Harbin | 47 (1.0) | 238 (2.1) | 374 (2.6) | 731 (2.5) | 1390 (2.3) |
| ALL | 4539 (100.0) | 11271 (100.0) | 14157 (100.0) | 29753 (100.0) | 59720 (100.0) |
